# BiomMiner: An advanced exploratory microbiome analysis and visualization pipeline

**DOI:** 10.1371/journal.pone.0234860

**Published:** 2020-06-18

**Authors:** Amirhossein Shamsaddini, Kimia Dadkhah, Patrick M. Gillevet

**Affiliations:** Microbiome Analysis Center, George Mason University, Manassas, Virginia, United States of America; University of Arizona, UNITED STATES

## Abstract

Current microbiome applications require substantial bioinformatics expertise to execute. As microbiome clinical diagnostics are being developed, there is a critical need to implement computational tools and applications that are user-friendly for the medical community to understand microbiome correlation with the health. To address this need, we have developed BiomMiner (pronounced as “biominer”), an automated pipeline that provides a comprehensive analysis of microbiome data. The pipeline finds taxonomic signatures of microbiome data and compiles actionable clinical report that allows clinicians and biomedical scientists to efficiently perform statistical analysis and data mining on the large microbiome datasets. BiomMiner generates web-enabled visualization of the analysis results and is specifically designed to facilitate the use of microbiome datasets in clinical applications.

## Introduction

Targeted amplicon-based analysis using 16S ribosomal RNA (rRNA) gene sequences is frequently used to explore complicated bacterial communities such as the human gut microbiome [1]. This approach has been used since 2007 for clinical diagnostics [2]. Comparative metagenomics has determined that there are three major ‘enterotypes’ affiliated with human gut microflora, and each of these enterotypes has a signatures genus, Bacteroides in the enterotype 1, Prevotella in the enterotype 2, and Ruminococcus in the enterotype 3 [3]. Another comparative metagenomics studies revealed a different gut microflora between ‘lean’ and ‘obese’ individuals [4]. Analysis of the large and complex bacterial communities like these studies demands sophisticated bioinformatics tools to efficiently process data in order to obtain a clear understanding of the dynamics of these ecological systems. There are several applications and pipelines available to process 16S rRNA gene sequencing data. The most popular open source packages are QIIME [5] and mothur [6]. Both QIIME and mother are all self-contained pipelines which can be used to analyze 16S rRNA gene sequencing data. Due to their comprehensive features and support documentation, QIIME and mother are considered the standard applications for microbiome analysis [7, 8]. As the microbiome field is rapidly expanding, demands for extra features and new more robust algorithm is high. Additionally, there is a need to making these packages more accessible to the clinical community. For instance, mothur and QIIME offer more than 100 individual commands and QIIME 2 has more than 15 commands and around 90 subcommands. Additionally, the installation of QIIME2 requires at least one hour using their private Bioconda [9] Channel on a high-end computer. Both QIIME and mothur documentation are detailed and include installation instructions with various tutorials that walking the reader step by step through a sequence of pipeline commands with example datasets to test their installation. While detailed documentation is helpful for research professionals, it can be overwhelming for the clinical users as they may not understand how modifications of complicated settings may alter the outcome and change the final analysis.

BiomMiner provides an advanced comprehensive data analysis workflow which covers both upstream and downstream analysis of 16S rRNA gene data sets. By eliminating the burden of command-line and step-by-step data processing, BiomMiner simplifies the processing down to single command and provides a standard HTML package of all generated downstream results with provenance logs for each step in the pipeline. This provides a simple mechanism to analyze microbiome data that is reproducible and easy to understand. This is critical to support clinical studies and the clinical diagnostics. BiomMiner offers the flexibility to choose between multiple Standard Operating Procedure (SOPs) such as mothur MiSeq SOP (https://www.mothur.org/wiki/MiSeq_SOP) for upstream data processing and provides a wide range of downstream statistical analysis with visualizations in a single HTML package. Sets of parameters are stored in JSON configuration files that can be used to reproducibly modify and re-run pipelines for evaluation and comparison using visualization within the HTML package. We provide documentation, installation instruction, example datasets, and case sample reports to facilitate rapid evaluation and adoption of the software under the MIT license at https://mbac.gmu.edu/mbac_wp/biomminer-readme/

## Approach

BiomMiner uses Snakemake [10] as the primary workflow management language for scalability and reproducible execution of various wrapper scripts developed in python and R for existing software tools. BiomMiner can easily redo failed steps and resume from checkpoints without repeating computationally intensive tasks which facilitates the testing of different parameters in a workflow. The other aspect of BiomMiner is the ability to deploy on both large clusters such as Amazon Web Services (AWS) or a single desktop computer with a few cores. BiomMiner generates an HTML package as standardized output using JavaScript to catalog all generated charts, graphs, and text-based result. Most of the results are visualized using ggplot2 [11] which can generate a high-resolution image with different formats. The user can open the HTML package on Internet browsing applications such as Chrome, Firefox, or Safari. The pipeline uses a single workflow configuration file (JSON config file) that can control most of the essential steps of the workflow and the users can easily modify them based on their research goals. At each step, BiomMiner keeps track of the logs generated in a specific directory that can be used to monitor the process.

## Results

BiomMiner utilizes many publicly available tools to perform the major steps of 16S rRNA analysis. Where necessary, we have written wrapper scripts to allow multiple samples to be run simultaneously and to integrate multiple tools by seamlessly converting file format. These scripts are typically written in either R or Python and are available at the BiomMiner tutorial link. BiomMiner workflow is divided into upstream and downstream pipelines. The upstream pipeline of the BiomMiner workflow follows the Schloss lab Standard Operating Procedure (https://www.mothur.org/wiki/MiSeq_SOP) for Illumina Miseq-SOP using mothur v1.34.

The downstream part of the BiomMiner workflow executes and visualizes the most popular statistical approaches for microbiome analysis such as alpha diversity, beta diversity, machine learning and generate an HTML file including results from downstream steps at the end of execution.

### BiomMiner upstream analysis modules

1. Paired read merging (assembly). If the data is generated by an Illumina instrument for example Illumina Miseq, read constructs are sequenced in both directions called “paired-end” read. BiomMiner merges pairs and creates one single read per pair generating a consensus sequence by aligning the forward and reverse reads and resolving any mismatches found in the alignment. This is accomplished by the “make.contigs” command in the mothur package.
2. Reducing sequencing and PCR errors. Raw reads that are generated by a next-generation sequencing machines such as 454 or Illumina have predicted error probabilities for each base indicated by quality (Q) scores. In many applications it is important to filter low quality reads to reduce the number of errors, especially in 16S rRNA gene sequencing experiments. The mothur “screen.seqs” command is used to filter out low-quality reads.
3. Chimera detection and removal. Chimeric sequences are an artifact formed from two or more different sequences joined together during PCR amplification. Chimeras are rare with shotgun sequencing but are common in amplicon sequencing when closely related sequences are amplified. The “chimera.vsearch” command is used to detect and discard chimeric reads.
4. Dereplication. The pipeline then discard duplicate sequences by running “uniq.seqs” command in the mothur package which compares every base in a sequence read and they must be identical over the full length of both sequences to be consider as duplicates.
5. Cluster the sequences into OTUs. We then use clustering algorithm (mothur opticlust) to create groups of closely related reads based on the similarity threshold (97% similarity) called operational taxonomic unit (OTU).
6. Assign taxonomic annotation to each OTU. We use RDP [12] v.16 as a reference in the command “classify.otu” to assign a consensus taxonomy for each OTU.
7. Create an OTU abundance table. OTU abundance tables are often stored as tabbed text files in which OTUs are rows and samples are columns. The abundance of an OTU is the number of reads derived from all biological sequences that are > = 97% identical to the OTU sequence. One entry in the table is usually a number of reads, also called a “count” or can be converted to relative abundance in the range 0.0 to 1.0.

### BiomMiner downstream analysis modules

**Fig 1:**
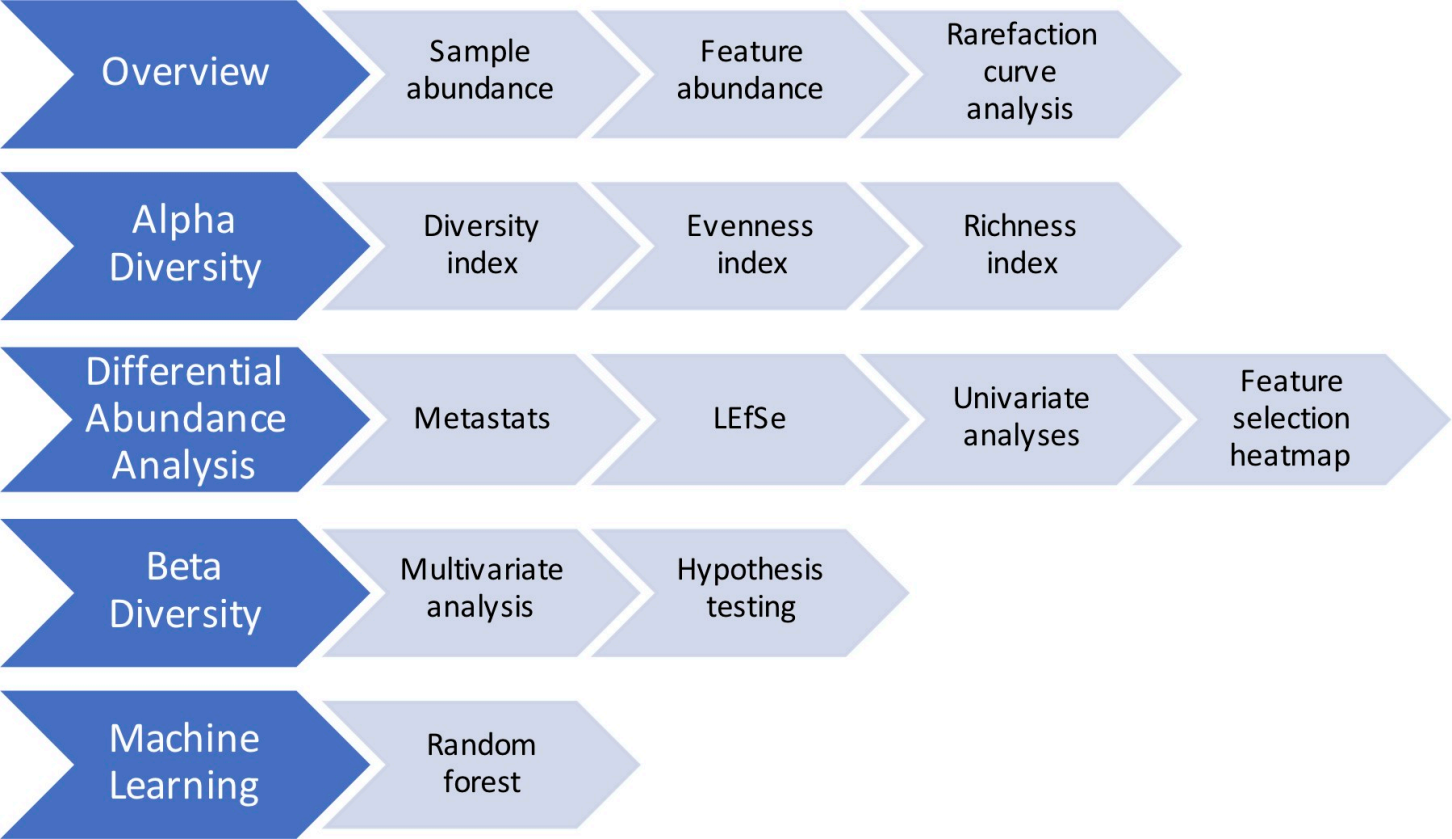
BiomMiner downstream analysis modules. There are five downstream analysis modules for BiomMiner that all start with the OTU abundance table as the input.

BiomMiner starts processing the OTU abundance table by generating a comprehensive HTML report including several most popular statistical approaches for microbiome analysis. These include an overview, alpha diversity, differential abundance analysis, beta diversity, and machine learning as shown in Fig 1.

A public benchmark from Schloss et al. 2012 [13] is selected here to represent the analysis pipeline and visualization features of BiomMiner. The selected benchmark is used to understand the effect of normal variation in the gut microbiome on host health. The study has been developed to determine whether there were significant changes in the murine gut microbiome community during the first year of life in Early (10 days following weaning) and Late (15 days following weaning).

### Overview

**Fig 2:**
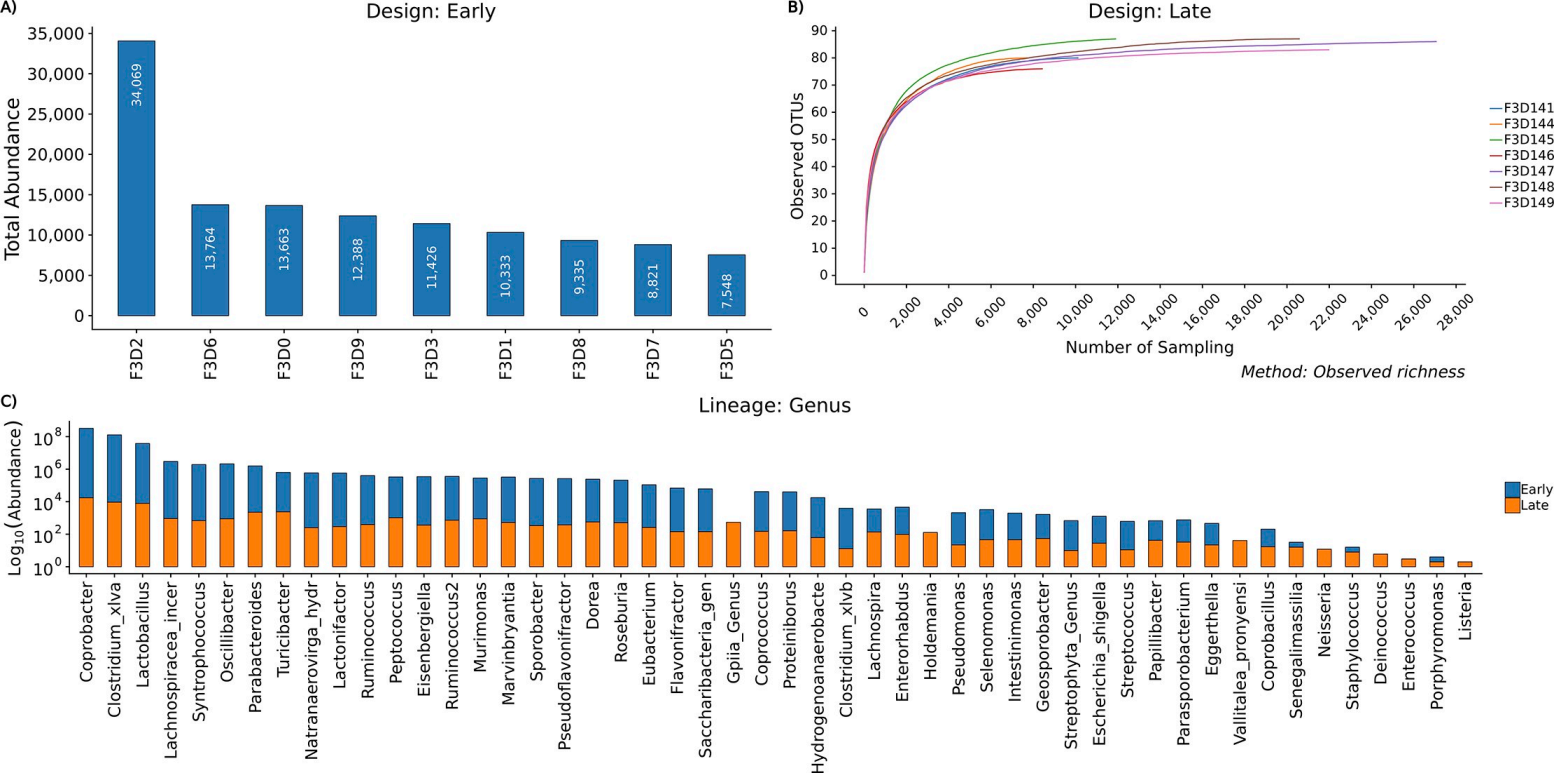
BiomMiner overview. Selected benchmark is used here to calculate and visualize the significant changes in the murine gut microbiome community in two state communities of the study, Early (10 days following weaning) and Late (15 days following weaning) [13]. This module produces total sample abundance bar plot, rarefaction curve, and groupwise feature abundance bar plot. Images here are from the study that is used as benchmark. (A) Total sample abundance bar chart for each community. Only the early group is shown here. The X-axis represents the sample name of the condition, and the Y-axis represents the total abundance of each sample.(B) Rarefaction curve plot. Only Late group is shown here. The rarefaction curve of the Late group reached an asymptote, which indicated that the sequencing depth was sufficient to represent the majority of species richness (observed richness). The X-axis represents number of samplings without replacement and the Y-axis represents the number of unique observed OTUs. (C) Total Feature Abundance bar chart. Log scaled comparison of the most abundant phylotypes between Early and Late group at the genus level. The X-axis represents Genus-level taxon and the Y-axis represents the abundance of each genus-level taxon on a log10 scale.

The main aim of the overview module is to provide a summary of the generated OTU abundance table like Groupwise sample abundance, feature abundance total count, and Rarefaction curve analysis. Groupwise sample abundance displays the total abundance of each sample for each Biological Condition as a Bar chart. Feature Abundance displays the total count of each OTU per each community which describes the distribution of OTU abundance per community. Rarefaction curve analysis, the estimate of sequencing depth and richness for each sample, is a very popular metric in microbiome analysis. BiomMiner uses mothur v.1.34 to perform rarefaction analysis. The goal of rarefaction is to determine whether sufficient observations have been made to get a reasonable estimate of a quantity that has been measured by sampling. The most commonly considered quantity is OTU richness (the number of different OTU in a group) (Fig 2).

### Alpha diversity

**Fig 3:**
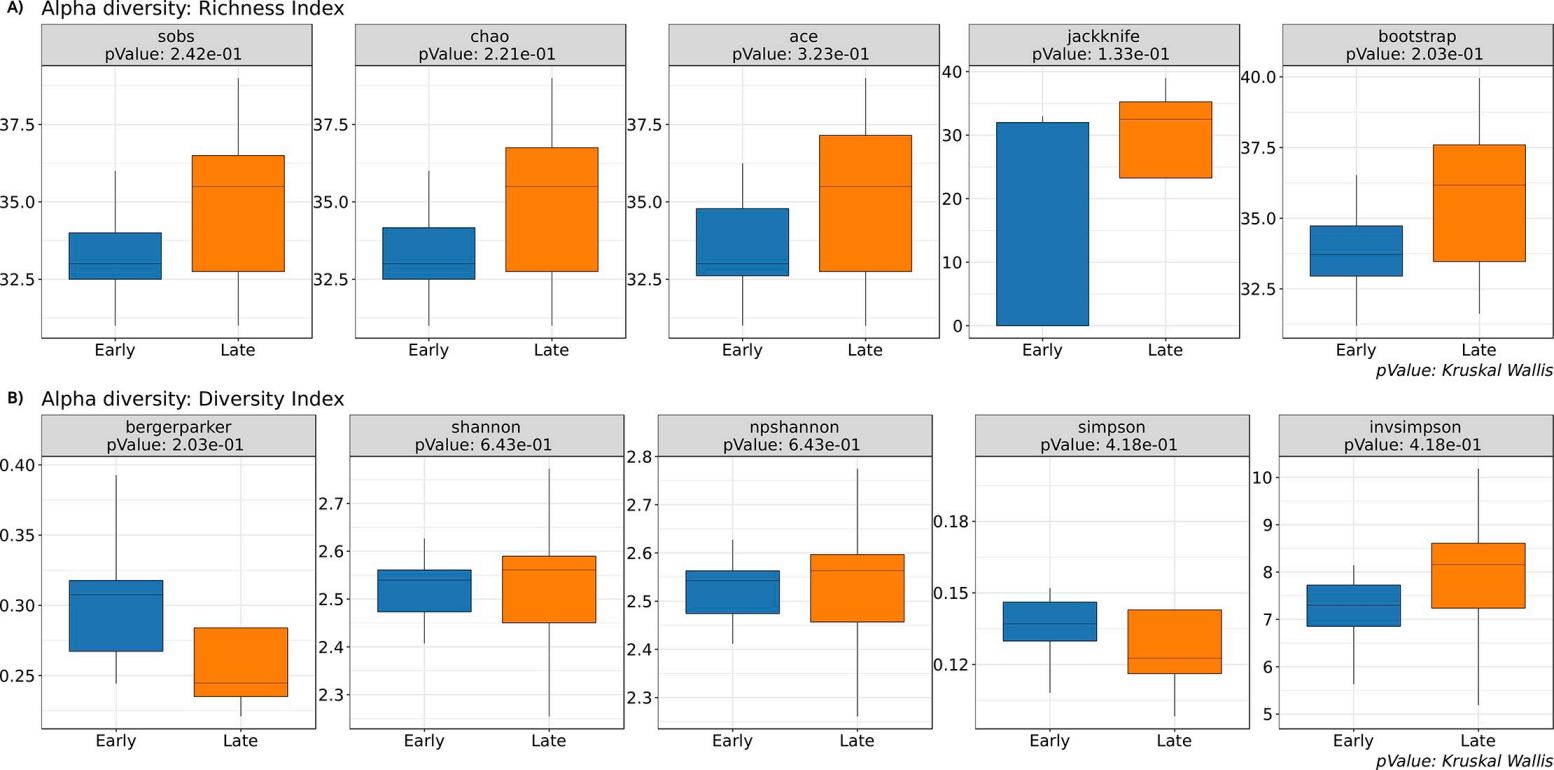
Alpha diversity. The selected benchmark is used here to calculate and visualize the significant changes in the murine gut microbiome community in the two states of the study, Early (10 days following weaning) and Late (15 days following weaning) [13]. Box and whiskers plots illustrate the median, quartiles, maximum and minimum of the alpha diversity value based on specific metrics. *pvalue* indicates significant difference between groups using Kruskal Wallis test. (A) Richness index boxplot. Richness index including sobs(Observed richness), Chao1 [17], ACE [18], Jackknife and bootstrap [19] were used to identify community richness differences between two groups. The X-axis represents biological condition and the Y-axis represents distribution of calculated richness index.(B) Diversity index boxplot. Diversity index including Berger-parker [16], Shannon, non-parametric Shannon [14], Simpson, and inverse-Simpson [15] were used to identify community diversity for diversity between subgroups of Early and Late category. Based on the box plots, there were no differences in community diversity between study subjects. The X-axis represents biological condition and the Y-axis represents the distribution of calculated diversity index.

Alpha diversity is the diversity within an individual sample. There are several alpha diversity indices available in BiomMiner to investigate diversity, richness and evenness such as Shannon [14], Simpson [15], Berger-Parker [16], and chao1 [17]. We are using mothur v.1.34 to calculate alpha diversity estimate. The richness estimate indicates the number of OTU found in a given sample regardless of how common or rare they are. The Evenness estimate indicates how evenly the richness (OTU count) is distributed. The Diversity estimate is a measurement of richness combined with evenness meaning it takes into account not only how many OTU is present but also how evenly distributed the numbers of each OTU are. After calculating Alpha diversity value of each population, BiomMiner then uses the calculated alpha diversity estimates in a statistical test to check whether the diversity, richness, and evenness between two conditions are significantly different by calculating the Bonferroni corrected *p*value of a Kruskal-Wallis test (Fig 3).

### Differential abundance analysis

BiomMiner also performs statistical methodology designed to identify differentially abundant features in metagenomic and 16S rRNA sequence datasets. We utilize well-established methods such as Metastats [20], LEfSe [21], and Kruskal–Wallis test available in mothur v.1.34. Metastats perform Fisher's exact test and calculate *pv*alue to provide a list of interesting features that are different between two groups. LEfSe (Linear discriminant analysis Effect Size) selects features (OTU) most likely to explain differences between communities by coupling a Kruskal–Wallis and a Wilcoxon rank-sum test) for statistical significance with a Linear Discriminant Analysis (LDA) to define the effect relevance.

**Fig 4:**
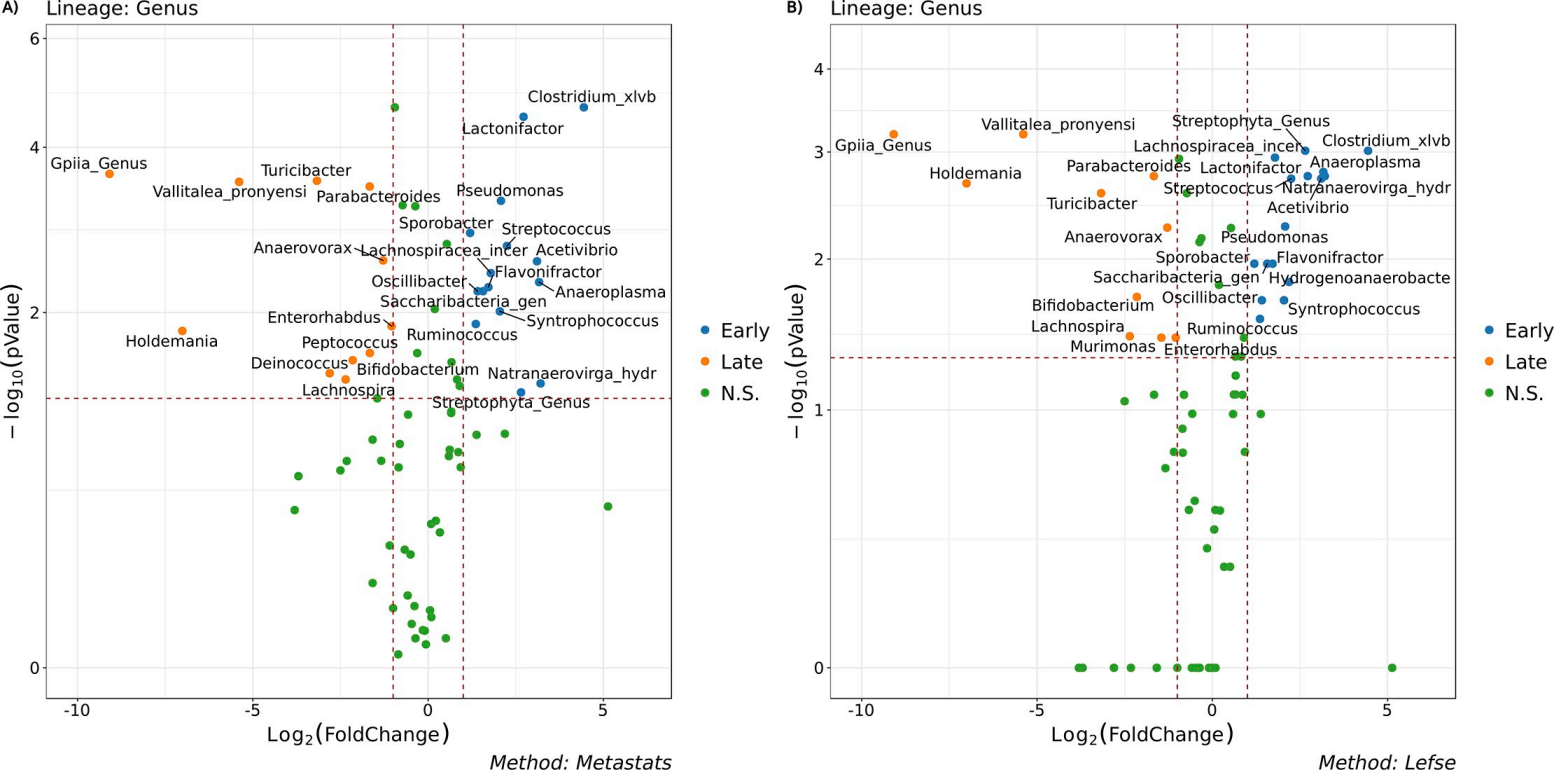
Differential abundance analysis: Volcano plot. The selected benchmark is used here to calculate and visualize the significant changes in the murine gut microbiome community in the two state communities of the study, early (10 days following weaning) and late (15 days following weaning) [13].Volcano plot showing OTU fold changes on X-axis and the negative logarithm (base 10) of the Bonferroni-adjusted *pvalue* on Y-axis. Dashed vertical and horizontal lines reflect the filtering criteria (fold change = ±1.0 and Bonferroni-adjusted *pvalue* > -log (0.05). Blue or Orange dots represent Genus entities that are significant based on Specific test (LEfSe or Metastats) at each group. The Green dots (N.S.) represent the Genus features either common between groups or classified as insignificant by the test (LEfSe or Metastats). In both A and B plots, the X-axis represents the abundance fold change on log2 scale, and the Y-axis represents the negative log10 of the calculated *pvalue*. N.S. means Non-significant. (A) Metastats Volcano plot suggest the differential features in metagenomic across two studies. (B) LEfSe Volcano plot could be interpreted as consistent difference in relative abundance of the analyzed fecal bacteria communities across the two groups.

Kruskal-Wallis (one-way ANOVA on ranks) is a non-parametric method for testing whether features originate from the same distribution between two communities. To quickly identify changes in large data, we used the “volcano plot” to present the result of each test. It is scatter plot which plots magnitude of the change(foldchange) of an OTU versus *pv*alue of the OTU from a statistical test on the X and Y-axis respectively, enabling quick visual identification of most the important features. To be considered as a significant feature; the foldchange value should be greater than 1 or less than -1, and the negative logarithm (base 10) of the *pv*alue should be above 1.13(-log (0.05)). We color each point based on their foldchange and *pv*alue so the user can easily pinpoint the biological and statistical significance of OTUs (Fig 4).

**Fig 5:**
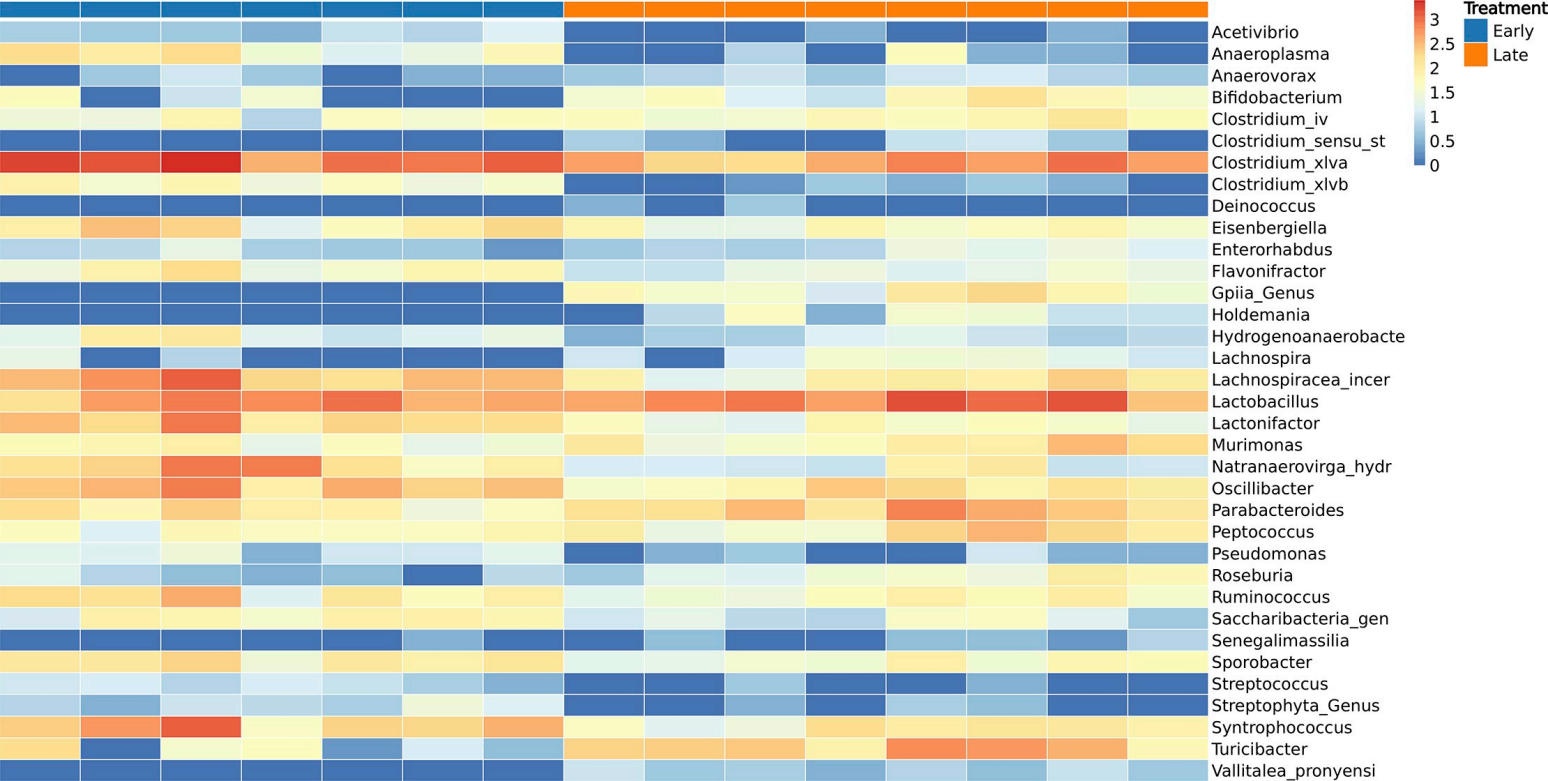
Differential abundance analysis: Heatmap. The selected benchmark is used here to calculate and visualize the significant changes in the murine gut microbiome community in two state communities of the study, early (10 days following weaning) and late (15 days following weaning) [13]. Heatmap showing the abundance variation of top 35 common bacterial taxa at the genus level which were significant OTUs (pvalue < 0.05) based on LEfSe, Metastats, and Kruskal-Wallis test. The rows represent the bacterial taxa and columns are the samples.

Since different statistical models sometimes produce *pv*alues that can be vastly different from each other, it is advisable to compare results from multiple methods and to visualize the features to gain more confidence. BiomMiner selects features by combining common significant features from each statistical test and then displays up to top 50 of these distinctive features in a heatmap plot. This implementation allows users to clearly pinpoint features of interest while minimizing the chance of missing important ones (Fig 5).

### Beta diversity: Ordination analysis

Ordination measurements are used to compare the similarity/ dissimilarity of the microbial communities. Microbiome studies are typically sparse with high-dimensionality, so it is hard to assess the direct correlation of microbiome composition with potential biological factors using OTUs abundances. Thus, ordination analyses is generally used to select a distance measurement method between groups and then conduct an analysis of the estimated distances [22]. BiomMiner utilize mothur v.1.34 for Beta diversity analysis. BiomMiner’s ordination module uses popular distance measure algorithms in microbiome studies like BrayCurtis [23], Jaccard [24], and weighted/ unweighted Unifrac [25] for performing ordination analysis and hypothesis testing to evaluate the dissimilarity of microbial community in each distance matrix.

**Fig 6:**
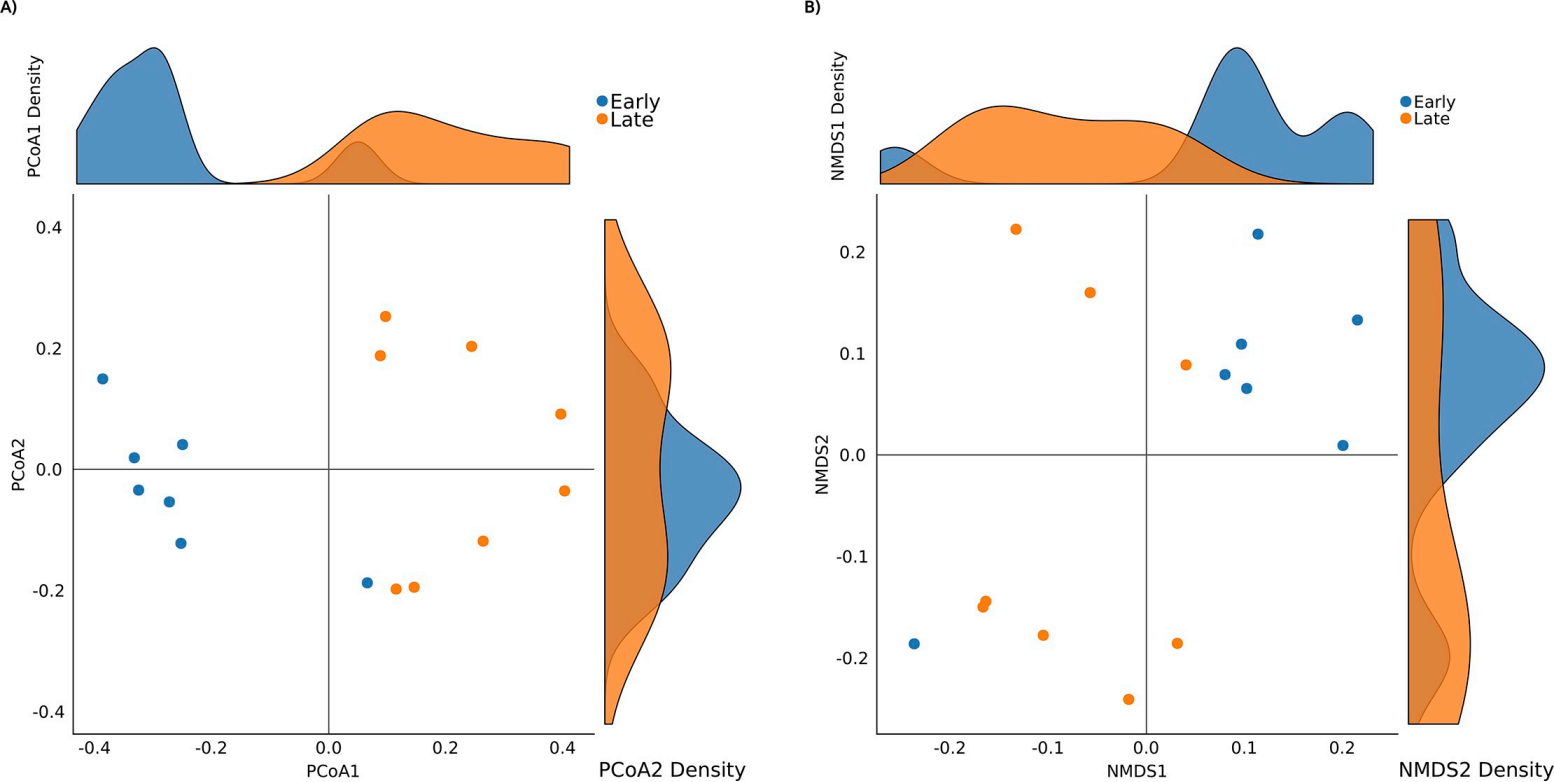
Beta diversity: Ordination analysis. The selected benchmark is used here to calculate and visualize the significant changes in the murine gut microbiome community in two state communities of the study, early (10 days following weaning) and late (15 days following weaning) [13]. Ordination analysis of microbial communities of the two groups was calculated using Bray-Curtis distance matrix and visualized using principal coordinate analysis (PCoA) (Plot A) and non-metric multidimensional scaling (NMDS) (Plot B). Points represent samples. Samples that are more similar to one another are ordinated closer together. The density plot on axis can be used to identify the similarity of distribution. The X-axis represent the first axis of the ordination while displaying the density of the sample’s ordination on first axis, and the Y-axis represent the second axis of the ordination while displaying the density of the sample’s ordination on second axis.

Ordination plots the distances between the communities into a Euclidean space and are then visualized via principal-coordinate analysis (PCoA) or non-metric multidimensional scaling (NMDS). Given a matrix of distances between samples, a PCoA visualizes these in a 2-dimensional Euclidian space represents their pair-wise distance in the original matrix. Non-metric multidimensional scaling (NMDS) is an indirect gradient analysis approach which produces an ordination based on a distance or dissimilarity matrix [26]. NMDS attempts to represent, as closely as possible, the pairwise dissimilarity between objects in a low-dimensional space (Fig 6).

### Beta diversity: Statistical hypothesis test

**Fig 7:**
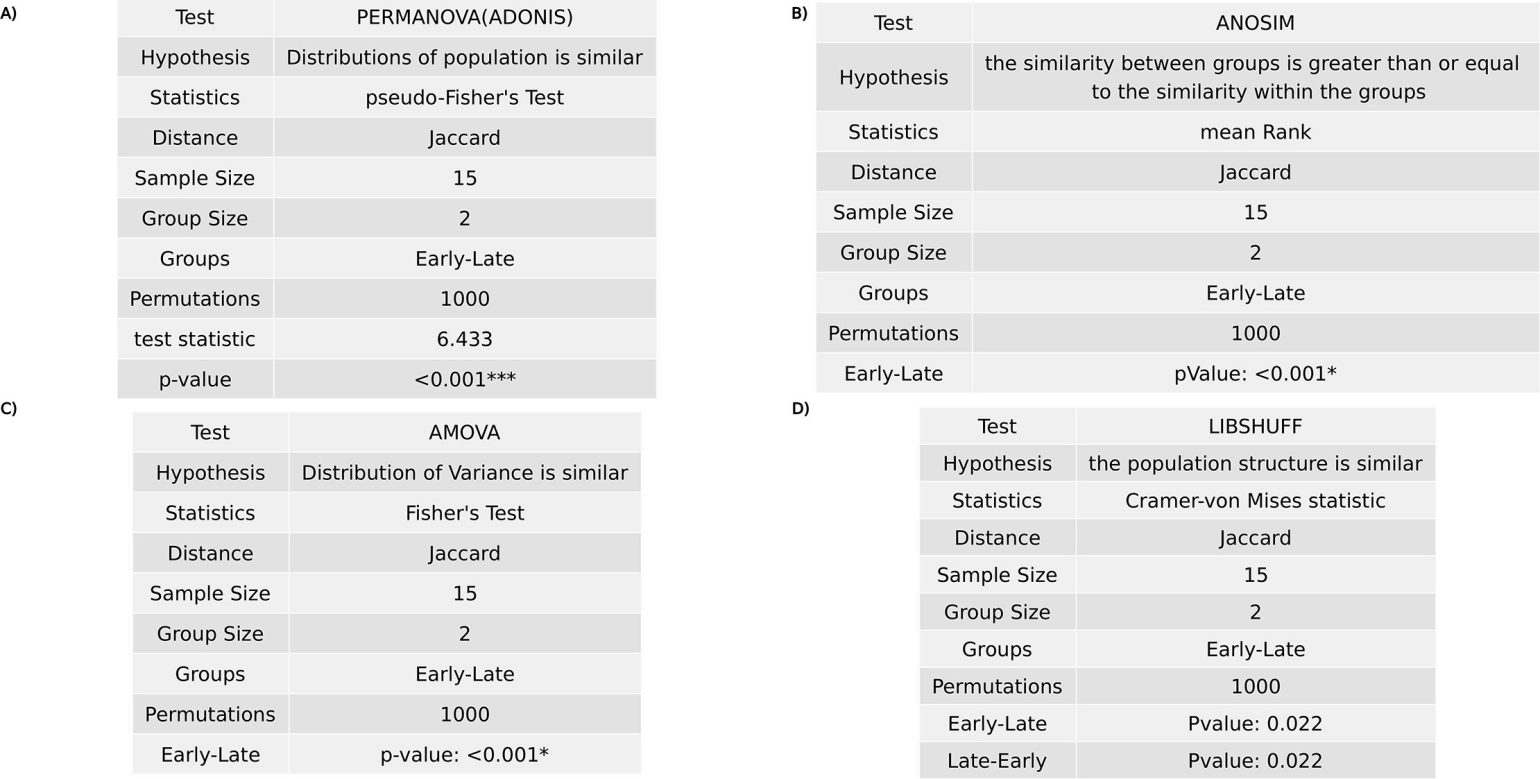
Beta diversity: Statistical hypothesis test. The selected benchmark is used here to calculate and visualize the significant changes in the murine gut microbiome community in two state communities of the study, early (10 days following weaning) and late (15 days following weaning) [13]. We used Jaccard distances as input for statistical hypothesis tests, comparing microbial community composition between two groups.(A) Permutational multivariate analysis of variance (PERMANOVA) compare microbial community and test the null hypothesis that distribution of microbial population is similar.(B) The Analysis of similarity (ANOSIM) is a nonparametric analog of traditional analysis of variance (ANOVA) and compares the mean of ranked dissimilarities between groups to the mean of ranked dissimilarities within groups.(C) Analysis of molecular variance (AMOVA) tests the variance of distribution between two groups.(D) LIBSHUFF describes whether two or more communities have the same structure using the Cramer-von Mises test statistic.

The Beta diversity’s statistical null hypothesis in microbiome studies is developed as “there is no difference of microbiome composition in experimental groups (e.g., healthy vs. patient)” or “there is no differences in distribution or structure of population of microbiome between cohorts”. BiomMiner uses most common approaches of microbiome hypothesis testing methods like AMOVA [27], HOMOVA [28], ANOSIM [29], LIBSHUFF [30], and PERMANOVA [31], and then displays the result of each test including the details of the test in separate table (Fig 7). The Analysis of similarity (ANOSIM) is rank-based or nonparametric version of analysis of variance (ANOVA) uses dissimilarity matrixes to provides a single *p*value indicating if community profiles (OTUs) similarity are significantly different between groups. PERMANOVA (Adonis) is a multivariate technique analogous to MANOVA and describes whether the variation in community OTU’s composition is different between groups. AMOVA (Analysis of Molecular Variance) can be used to measure the apportionment of OTU variance between pairs of populations [32]. LIBSHUFF describes whether two or more communities have the same OTU structure, different, or subsets of one another using the Cramer-von Mises test statistic. Homogeneity of molecular variance (HOMOVA) determines whether the diversity of features (OTUs) in each community is significantly different.

### Machine learning

**Fig 8:**
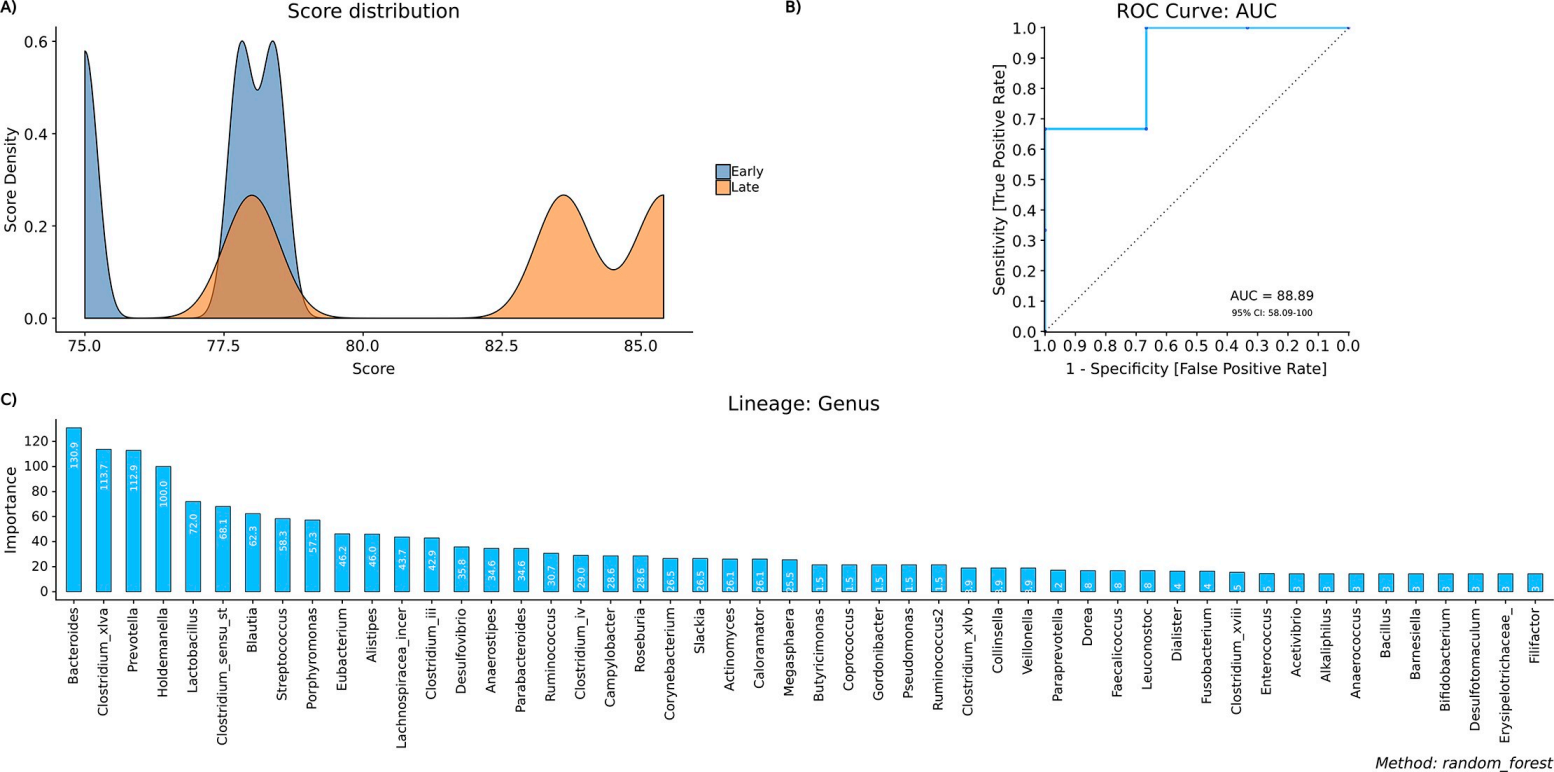
Machine learning module. The selected benchmark is used here to calculate and visualize the significant changes in the murine gut microbiome community in two state communities of the study, early (10 days following weaning) and late (15 days following weaning) [13]. (A) Score distribution gives us visual information on skewness, distribution and our model’s facility to distinguish each class. Here we can see how the model has distributed both our categories, (the more separate, the better). The X-axis represent the distributions of calculated prediction score and the Y-axis represent the sample prediction score density. (B) Receiver operating characteristic (ROC) curves for Random Forest classifier. The ROC curve will give us an idea of how our model is performing with our test set. The ROC curve is plotted with True Positive Rate (TPR) against the False Positive Rate (FPR) where TPR is on Y-axis and FPR is on the X-axis. if the AUC is close to 50% then the model is as good as a random selector. On the other hand, if the AUC is near 100% then you have a “perfect model”. (C) Important feature. It shows the importance of each feature by calculating the increase in the model’s prediction error after permuting the feature. A feature is “important” if shuffling its values increases the model error as the model relied on the feature for prediction accuracy. A feature is “unimportant” if shuffling its values leaves the model error unchanged as the feature did not contribute to the prediction accuracy.

In addition to the standard statistical approaches mentioned above, BiomMiner also supports a number of machine learning approaches for supervised learning and feature selection, such as random forest (RF) and support vector machine (SVM). In many recent reports on the classification of microbiome data, it has been shown that machine learning and data mining have performed well [33, 34]. BiomMiner uses the Caret [35] R package to calculate Random forest (RF) and SVM and uses the “predict” R package modeling algorithm on the test set. When running machine learning in BiomMiner we used 70% of OTU abundance table as the training set to train the model and to evaluate the performance of the generated model and the remaining 30% of the OTU abundance table as a test set. In order to assess the performance of the machine learning model, we plot the Receiver operating characteristic (ROC) curve, the predicted score distributions density plot, and the important feature bar plot. The Receiver operating characteristic (ROC) curve indicates how well our model is performing with our test set. It tells how much of the model is capable of distinguishing between communities. The ROC curve is plotted with True Positive Rate (TPR) against the False Positive Rate (FPR) where TPR is on Y-axis and FPR is on the X-axis. The predicted score distributions density plot gives us visual information on skewness, distribution, and our model's accuracy to distinguish each class. Important feature bar plot shows the impact of each feature (OTU) on the accuracy of the model. A feature (OTU) is "important" if shuffling its values increases the model error indicating that the model relied on the feature for the prediction. Clearly, for unimportant OTU, the permutation of its value will have little to no effect on the model accuracy (Fig 8).

## Discussion

Several excellent web-based or desktop applications have been developed over the past decade to support microbiome data analysis. Most of these tools have been developed primarily for raw sequence processing, clustering, and annotation, with limited or yet in development support for advanced statistical analysis and visual exploration. Other applications only focused on the downstream portion of the analysis and let the user upload their processed data which suffer from several issues like format incompatibility, unsupported annotation (which may lead to garbage in, garbage out patterns). BiomMiner complements these applications by providing complete upstream analysis and comprehensive support for statistical, visual, and meta-analysis on the downstream side of the experiment. While developing BiomMiner, we aimed to create a sophisticated yet easy to understand platform for microbiome data analysis. Users can easily download the analysis result at high-resolution images that generated on BiomMiner for using in their publications or they can import text-based results from BiomMiner into other software for further analyses. The future advancement of BiomMiner will focus on integrating new downstream analysis such as functional genomics.

## Supporting information

S1 File Summary of comparison between BiomMiner and currently available software. This PDF file contains a table summarizing a comparison of supported capabilities between BiomMiner, phyloseq [1], QIIME and mothur. A ‘‘1” or ‘‘0” indicates that the capability is supported or not supported, respectively. “T” means the result is available as text format file and “G” indicate the generated file is graphic based result and “T/G” mean the result is available in text and graphic based format. This is not a comprehensive summary of the capabilities of each package, but rather the capabilities of relevance to this article.
(PDF)

S1 Data (DOCX)
