# Supplementary material for: BiomMiner: An advanced exploratory microbiome analysis and visualization pipeline
Source: PLoS One. 2020 Jun 18;15(6):e0234860. doi: 10.1371/journal.pone.0234860 (PMC7302521; doi:10.1371/journal.pone.0234860)
Supplement: S1 File — This PDF file contains a table summarizing a comparison of supported capabilities between BiomMiner, phyloseq [1], QIIME and mothur. A ‘‘1” or ‘‘0” indicates that the capability is supported or not supported, respectively. “T” means the result is available as text format file and “G” indicate the generated file is graphic based result and “T/G” mean the result is available in text and graphic based format. This is not a comprehensive summary of the capabilities of each package, but rather the capabilities of relevance to this article. (PDF) [file pone.0234860.s001.pdf]

Category	subCategory	BiomMiner	phyloseq	QIIME	QIIME2	mothur
Upstream data analysis						
	Sequence processing	1	0	1	1	1
	upstream QC	1	0	0	1	0
Overview						
	Taxonomy composition	T/G	G	T/G	T/G	T
	Sample abundance	T/G	G	T/G	T/G	T
	Rarefaction analysis	T/G	0	T/G	T/G	T
ALPHA diversity						
	Richness	T/G	G	T/G	T/G	T
	Eveness	T/G	G	T/G	T/G	T
	Diversity	T/G	G	T/G	T/G	T
Differential abundance analysis						
	Lefse	T/G	0	0	0	T
	Metastats	T/G	0	0	0	T
	Kruskall-Wallis	T/G	0	T	T	T
BETA diversity: Ordination						
	PCoA	T/G	G	T/G	T/G	T
	NMDS	T/G	G	T/G	T/G	T
BETA diversity: Hypothesis testing						
	ANOSIM	T/G	0	T	T	T
	AMOVA	T/G	0	0	0	T
	HOMOVA	T/G	0	0	0	T
	LIBSHUFF	T/G	0	0	0	T
	PERMANOVA	T/G	0	T	T	0
Machine learning						
	prediction score ROC Curve	T/G	0	0	0	0
	Important Feature selection	T/G	0	0	0	0
	prediction score distribution	T/G	0	0	0	0

1	Available
0	Not Available
T	Text based result
G	Graphic based result
T/G	Text and Graphic based result
