# Supplementary material for: BiomMiner: An advanced exploratory microbiome analysis and visualization pipeline
Source: PLoS One. 2020 Jun 18;15(6):e0234860. doi: 10.1371/journal.pone.0234860 (PMC7302521; doi:10.1371/journal.pone.0234860)
Supplement: S1 Data — (DOCX) [file pone.0234860.s002.docx]

**Supporting information references**

1. McMurdie PJ, Holmes S. Waste Not, Want Not: Why Rarefying Microbiome Data Is Inadmissible. PLoS Computational Biology. 2014;10(4):e1003531. doi: 10.1371/journal.pcbi.1003531.
